# Visual-Text Reference Pretraining Model for Image Captioning

**DOI:** 10.1155/2022/9400999

**Published:** 2022-01-21

**Authors:** Pengfei Li, Min Zhang, Peijie Lin, Jian Wan, Ming Jiang

**Affiliations:** Hangzhou Dianzi University, Baiyang Road #2, Hangzhou, China

## Abstract

People can accurately describe an image by constantly referring to the visual information and key text information of the image. Inspired by this idea, we propose the VTR-PTM (Visual-Text Reference Pretraining Model) for image captioning. First, based on the pretraining model (BERT/UNIML), we design the dual-stream input mode of image reference and text reference and use two different mask modes (bidirectional and sequence to sequence) to realize the VTR-PTM suitable for generating tasks. Second, the target dataset is used to fine tune the VTR-PTM. To the best of our knowledge, VTR-PTM is the first reported pretraining model to use visual-text references in the learning process. To evaluate the model, we conduct several experiments on the benchmark datasets of image captioning, including MS COCO and Visual Genome, and achieve significant improvements on most metrics. The code is available at https://github.com/lpfworld/VTR-PTM.

## 1. Introduction

Pretraining models based on large-scale data greatly improve the performances of cross-modal understanding tasks, in which these models are also considered as the mainstream methods to fine tune the downstream tasks (e.g., image-text retrieval, visual question answering, and video question answering) [1–5]. However, it is not possible to directly use the pretraining models for the generation tasks. On the one hand, the pretraining models with BERT as the benchmark models only use the encoder of the transformer. If the generation task is preferred, a decoder needs to be provided [6–8]. On the other hand, the mask matrix used by the pretraining models for training is mostly designed for understanding tasks but not for the cross-modal generation tasks [9, 10]. We focus on a unified representation of the encoder and decoder and provide a mask design for the cross-modal generation tasks.

Typically, the input to a pretraining model includes words in the text, regions of interest in an image, and markers with special meanings. It is common to aggregate and align visual features and text features through an attention mechanism before input operation [5, 8, 9]. However, this method cannot make the model refer to the visual and text features of the image at all times in the learning process. In the process of adjusting the visual pretraining model, the natural language features extracted by LSTM are used as a reference to help the visual pretraining model better identify the image content [11–13]. Inspired by this observation, we propose that visual and textual features can be used as references for pretraining models to help them generate captions better.

**Figure 1:**
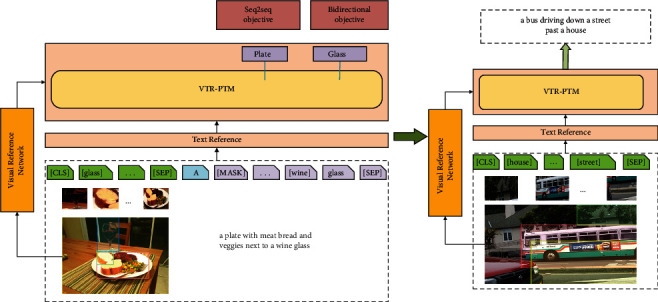
The left side is the pretraining process of VTR-PTM, and the right side is to fine tune the trained model for image captioning.

In this paper, we propose the Visual-Text Reference Pretraining Model (VTR-PTM), which is used for image captioning (see Figure 1). Specifically, VTR-PTM uses the unified encoder-decoder representation architecture and integrates the visual reference module into the encoder-decoder architecture consisting of 12 layers of transformer blocks, each having a masked self-attention layer and feed-forward network. The unified modeling is achieved by employing a shared transformer network and utilizing specific self-attention masks to control what context the prediction conditions on. The model is pretrained on a mass of image-text pairs and modeled for bidirectional objectives and sequence-to-sequence objectives. During the pretraining process, we use the Faster RCNN [14] to detect the image and take the results as the key text reference. They will be input into the VTR-PTM in combination with captioning and special markers. Meanwhile, we use the ResNet101 [15] to extract image features and encode the image features as the visual reference. They are added to the existing and unconditional *g* (gain) and *b* (bias) of layer normalization (LN) in transformers in the form of incremental parameters. The pretraining model constantly refers to the information from the image and key text, emphasizes the feature selection related to the image captioning, and reduces the weight of irrelevant features to achieve accurate captioning of the image. We have achieved promising results on two challenging benchmark datasets (MS COCO [16] and Visual Genome [17]), and the corresponding ablation experiments also prove the effectiveness of VTR-PTM.

In summary, our main contributions include the following three aspects:

1. We propose a new pretraining model VTR-PTM with a unified encoder-decoder representation with the bidirectional objective and the sequence-to-sequence objective so that the pretraining model can be applied to the generation tasks.
2. We introduce the concept of visual-text reference for the pretraining model. In the process of pretraining model learning, constantly referencing visual-text features from images helps the model generate captions better.
3. We perform image captioning tasks on MS COCO and Visual Genome datasets with the trained VTR-PTM and achieve promising results.

## 2. Related Work

### 2.1. Image Captioning

Image captioning is a comprehensive task combining the fields of computer vision and natural language processing. This task is challenging, and it needs to accurately find the salient objects in an image, the properties of the objects, the relationship between objects and the scene, and correct articulation in natural language. In the early years, operators were used to extract the features of an image and obtain the possible targets and their attributes by SVM. Then, CRF or some rules were considered to generate captions of an image [18, 19]. Through machine translation, the encoder-decoder structures were used for image captioning, which encode images by a convolutional neural network and decode the encoding features by the recurrent neural network [20, 21]. After the introduction of the attention mechanism, performances have been further improved [22, 23]. In addition, some methods establish relationships among the objects in an image and use them to construct a scene graph. By analyzing the scene graph, image captions are obtained [24–27]. Thanks to the success of the models such as transformer [28] and BERT in the field of natural language processing, studies on image captioning have been further advanced. NG-SAN [29] proposes a normalized self-attention mechanism and demonstrates the benefits of internal normalization. To address the transformer's inability to model the geometry of the input objects, geometric awareness-based self-attention is proposed, which enables to clearly and effectively consider the relative geometric relations among the objects in the images. U-VLP [7] proposes a unified visual language pretraining model, which uses two unsupervised learning objectives of bidirectional and sequence-to-sequence to conduct unified pretraining for lots of image-text pairs and to fine tune the pretraining model to adapt to the downstream tasks.

### 2.2. Pretrained Language Models

The pretraining models trained on a large corpus can learn common language representations and help complete downstream tasks. The pretraining models provide a better model initialization, which can bring in a better generalization performance and accelerate the convergence speed of the target task. A detailed introduction of the pretraining models is provided in literature [27]. Here, we provide a specific review related to our model. BERT is of great significance to NLP, which is mainly based on two core ideas: transformer architecture and unsupervised learning. It increases the generalization ability of the word vector model and fully describes the features at the character level, word level, sentence level, and even at the level for intersentence relationships. Unified Language Model Pretraining for Natural Language Understanding and Generation (UNILM) [30] also proposes a pretraining method, which is very simple and directly multiplexes the structure and parameters of BERT. Specifically, it uses a shared multilayer transformer that is pretrained with three language modeling goals: one way, bidirectional, and sequence to sequence. Each target specifies a different binary value in the self-attention mask to control the content available to the language model. Both BERT and UNILM can be fine-tuned to handle a variety of downstream tasks. BERT is mostly used in natural language understanding tasks, while UNILM aggregates context for different types of language models by configuring different self-attention masks. Therefore, UNILM can be used in both the natural language understanding task and the natural language generation task.

### 2.3. Cross-Modal Pretraining Models

According to the model architectures, the solutions to the cross-modal pretraining models can be divided into one-stream models and two-stream models. The one-stream models integrate text information and visual information into combined models, while the two-stream models process text and visual information, respectively. Video BERT [5] processes video-language tasks using BERT, which changes BERT processing “sentence pair” to “sentence-video pair,” and then masks the video frame randomly. Similar to Video BERT, Visual BERT [31] serializes text features and visual features. The difference is that its visual features use region features without a mask. Unicoder VL [7] also fuses data at the beginning of training. The difference is that it uses Faster RCNN to process images and encodes the extracted image regions and positions. VL BERT [9] uses regions as visual features and adds the features of the complete image to the input part of the model. In ViL BERT [3], images and texts are sent into two different streams into the co-attentional transformer layer, where images are generated by Faster R-CNN to extract features from candidate regions to generate embedding, while texts are generated by several additional transformer layers. LXMERT [4] uses a two-stream model. The texts and the images are encoded separately through an independent coding layer, and then, the texts and the images are aligned and fused semantically through a modal interaction coding layer.

The above studies use datasets of different sizes to adjust the pretraining model during pretraining. The pretraining process is based on large datasets and self-supervised learning [3–5, 7, 9, 31]. Some studies on the combination of semisupervised learning and pretraining model give us some new enlightenment [32–36]. Semisupervised learning is a learning method that uses annotated and unannotated data comprehensively, which can reduce the workload of manual annotation data and bring high accuracy. It is widely used in image classification, text classification, and other fields [32–34]. Sun et al. [35] studied how to combine semisupervised learning and pretraining models more effectively, that is, how to make better use of annotated and unannotated corpora in large-scale domains under the premise of large-scale general domain pretraining so as to maximize the effect of the model. Chen et al. [36] attempted to use the typical paradigm of semisupervised learning on the ImageNet dataset with superior results.

## 3. Approach

In this section, we first introduce the architecture of VTR-PTM, including preprocessing details for images and texts, and adjustments to LN layers in the transformer layers of the pretraining model. Then, pretraining objectives including bidirectional and sequence-to-sequence are introduced.

### 3.1. Visual-Text Reference Pretraining Model

Our cross-model transformer network unifies the transformer encoder and decoder into a single model. Its backbone is the same as the BERT base. In contrast to the existing pretraining model, we design a dual-stream input mode for image reference and text reference, and we design a novel LN layer for transformer, called “VRLN” (Visual Reference Layer Normalization).

#### 3.1.1. Text Reference

The content of the text reference is different during model training and testing. During training, the text reference includes the results of object detection, target captions, and special markers. During testing, only results from object detection are included. We define the input image as *I* and the target sequence as *S*. We use the pretrained Faster RCNN [14] on Visual Genome [17] to detect objects in the images and obtain *n* object detection boxes *B*={*b*_*i*_,  *i*=1,…,  *n*}. The corresponding region label is *C* =  {*C*_1_, *C*_2_,…, *C*_*n*_}, *C*_*i*_ ∈ ℝ^*d*^. *C*_*i*_ represents the category of the final prediction, and *d* represents the total number of the prediction categories. The calculation process is shown in as follows:

(1) $$\begin{array}{c} B=\text{ Faster RCNN}\left(I\right), \end{array}$$

(2) $$\begin{array}{c} X=\text{ResNet}\left(B\right), \end{array}$$

(3) $$\begin{array}{c} C=\text{SoftMax}\left(X\right). \end{array}$$

The special markers include [CLS], that is before the first keyword the representation vector obtained by BERT used for subsequent classification tasks, [SEP] used to separate two input sentences or to indicate the end of a sentence, and [MASK] used to cover some words in the sentence. After covering the words with [MASK], the [MASK] vector output by BERT is used to predict what the words are. For example, (see Figure 1), we get the keyword set *C* ={glass,bread,plate}. *C* is combined with target caption and special markers as the input sequence ([CLS] glass bread plate [SEP] a [MASK] with meat bread and vegetable next to a wine [MASK] [SEP])

#### 3.1.2. Visual Reference

**Figure 2:**
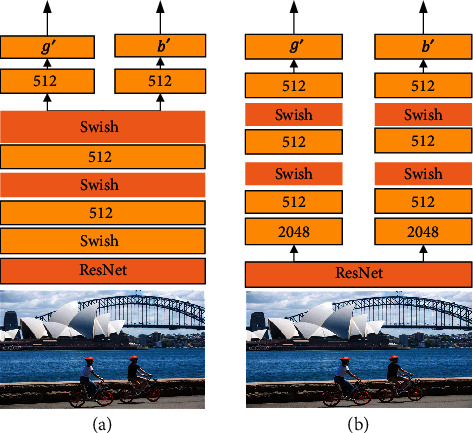
Two different visual reference networks: (a) single-channel visual reference network and (b) dual-channel visual reference network.

The content of the visual reference is obtained by coding the image through the visual reference network. For a given image *I*, we adopt two different encoding methods, i.e., the single-channel visual reference network (VRN-SC) and the dual-channel visual reference network (VRN-DC) (see Figures 2(a) and 2(b)).

Our backbone network uses the pretrained ResNet on ImageNet to obtain the image feature *I*_resnet_, with a dimensionality of 2048. The process is formulated as

(4) $$\begin{array}{c} I_{\text{resnet}}=\text{ResNet}\left(I\right). \end{array}$$

In the single-channel visual reference network, *I*_resnet_ is encoded to 512-dimensional vectors through two fully connected networks and two Swish layers, and then up to 768-dimensional vectors to ensure that it can be input into transformer as increments (e.g., in Figure 2(a)), where *g*′ and *b*′ are expressed as

(5) $$\begin{array}{c} g^{\prime }=b^{\prime }\\ =f_{\text{VRN}-SC}\left(I_{\text{resnet}}\right). \end{array}$$

In the dual-channel visual reference network, the calculation process of *I*_resnet_ is the same as that of the single-channel visual reference network, but the coding of *g*′ and *b*′ is independent (e.g., in Figure 2(b)), and their expressions are

(6) $$\begin{array}{c} g^{\prime }=f_{\text{VRN}-DC1}\left(I_{\text{resnet}}\right),\\ b^{\prime }=f_{\text{VRN}-DC2}\left(I_{\text{resnet}}\right), \end{array}$$

where *f*_VRN−*DC*1_ and *f*_VRN−*DC*2_ represent two different columns. Although the calculations of *I*_resnet_ are the same, the adjustment of the two columns of the parameters is gradually different in the training process of the model, and the final *g*′ and *b*′ are different. In the ablation experiments, we also compare the effects of these two coding methods.

#### 3.1.3. Visual Reference Layer Normalization

Layer normalization can normalize the results of a hidden layer in a neural network into standard normal distribution to accelerate convergence. Its input is a matrix *X*, and the mean *μ* and variance *σ* are calculated by the column unit of the matrix:

(7) $$\begin{array}{c} \mu_{j}=\frac{1}{m}\sum_{i=1}^{m}x_{ij},\\ \sigma_{j}^{2}=\frac{1}{m}\sum_{i=1}^{m}{\left(x_{ij}-\mu_{j}\right)}^{2}, \end{array}$$

where *i* is the row of *X*, *j* is the column of *X*, and*ε* is a small decimal to prevent the denominator from being 0. The normalized value can be obtained by subtracting the mean value of this column from each element of each column and dividing it by the standard deviation of this column. The output of LN is

(8) $$\begin{array}{c} \text{Layer Norm}\left(x\right)=\frac{x_{ij}-\mu_{j}}{\sqrt{\sigma_{j}^{2}+\epsilon }}. \end{array}$$

In LN, we also need a set of parameters to ensure that the normalization does not destroy the existing information. The process is formulated in equation (9). There are already readymade, unconditional, fixed length *g* (gain) and *b* (bias) (equivalent to *γ* and *β* in BN).

(9) $$\begin{array}{c} f_{LN}\left(X\right)=g*\text{Layer Norm}\left(X\right)+b. \end{array}$$

VRN encodes image features into the same dimensions as those of *g* and *b* and then adds the two coding results *g*′ and *b*′ to *g* and *b* in the form of increments, respectively. To prevent disturbing the original pretraining weight, the two transformation matrices can be zero initialized so that in the initial state, the model remains consistent with the original pretraining model.

(10) $$\begin{array}{c} \hat{g}=g+g^{\prime },\\ \hat{b}=b+b^{\prime }. \end{array}$$

**Figure 3:**
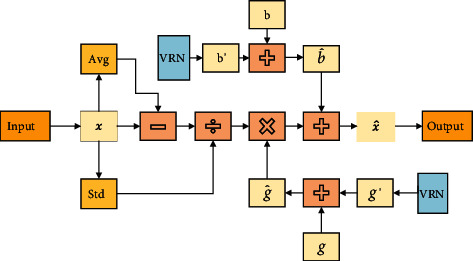
Calculation process of VRLN.

Then, the features can be obtained from VRLN:

(11) $$\begin{array}{c} \hat{X}=f_{\text{VRLN}}\left(X\right)=\hat{g}*\text{Layer Norm}\left(X\right)+\hat{b}, \end{array}$$

where *X* is the output of the previous model. The calculation process is shown in Figure 3.

### 3.2. Pretraining Tasks

Our pretraining process refers to U-VLP [8] and UNILM [30], using the design of the two different language objectives for VTR-PTM generation. In BERT, 15% of the input text is replaced by [MASK], random text, or original text, and the replacement probabilities are 80%, 10%, and 10%, respectively. We adopt similar replacement principles. We input the corresponding output vector calculated by the model into SoftMax to predict possible words. The only difference between the two language objectives is the self-attention mask. The input to the first transformer is defined as *H*^0^, as shown in the following equation:

(12) $$\begin{array}{c} H^{0}=\left[C_{\left[\text{CLS}\right]},C_{1},\;\ldots ,C_{n},\ldots ,y_{\left[\text{SEP}\right]},y_{1},\ldots ,y_{T},y_{\left[\text{SEP}\right]}\right]\in {\mathbb{R}}^{d*U}, \end{array}$$

where *U* = *N* + *T* + 3, N is the number of keywords, and *T* is the length of the target caption. The output of the encoder at different layers of the L-layer transformer can be expressed as *H*^*l*^, as shown in the following equation:

(13) $$\begin{array}{c} H^{l}=\text{Transformer}\left(H^{l-1}\right),\;l\in \left[1,L\right]. \end{array}$$

We define the mask matrix of self-attention as *M* ∈ ℝ^*U∗U*^, which is shown in the following equation:

(14) $$\begin{array}{c} M_{jk}=\left\{\begin{array}{cc} 0, & \text{allow to attend,}\\ -\infty , & \text{prevent from attending,} \end{array}\right. \end{array}$$

where *M*_*jk*_ represents the elements in the mask matrix, *j*, *k* ∈ [1,…, *U*]. The self-attention head can be expressed as *A*^*l*^, and the calculation process is shown in the following equation:

(15) $$\begin{array}{c} A^{l}=\text{softmax}\left(\frac{Q^{T}K}{\sqrt{\text{d}}}+M\right)V^{T}, \end{array}$$

(16) $$\begin{array}{c} V=W_{V}^{l}H^{l-1},\\ K=W_{K}^{l}H^{l-1}, \end{array}$$

where *W*_*V*_^*l*^, *W*_*V*_^*l*^, and *W*_*K*_^*l*^ are the embedding weights. The intermediate variables *V*, *Q*, and *K* indicate values, queries, and keys. *A*^*l*^ is further encoded by the feedforward layer and combined with the residual network to form the output *H*^*l*^.

The bidirectional objective: the model allows all tokens to focus on each other in the prediction and encodes the context information of the left and right directions of the masked token in the training process. The operation is to set *M* in equation (14) to a zero matrix, and each token is allowed to appear in all the locations in the input sequence, indicating that the global information can be involved in the prediction of the token.

The sequence-to-sequence objective: the input of this model consists of the source segment and the target segment. During the training process, tokens in the two segments are randomly masked off, and then, the model is asked to predict the words masked off. In the source segment prediction process, mutual attention is allowed from two directions in the source segment, while in the target segment prediction process, only the left context in the target segment, the current word, and all the tokens in the source segment can be used. In this way, the model can implicitly learn a bidirectional encoder and a unidirectional decoder.

### 3.3. Fine Tuning for Image Captioning

We fine tune the pretrained VTR-PTM on the target dataset using the sequence-to-sequence objective and use beam search for the generated sequence. If not specifically specified, the beam size is 3. We use [CLS] as the starting flag, use [CLS] and [SEP] to mark the keywords, and then start the generation by inputting [MASK] and using word likelihood. The [MASK] in the previous input sequence is replaced with the generated word, and the new [MASK] is attached to the input sequence to trigger the next prediction. When [SEP] is selected, the generation terminates.

## 4. Experiments and Results

### 4.1. Experimental Setup

#### 4.1.1. Datasets

We conduct pretraining on Flickr30K [37]. The dataset includes 31,783 images, each of which has the corresponding 5 descriptions. Compared with large datasets such as Conceptual Captions [38], our pretraining process uses a smaller cost. To evaluate the performance of the model proposed in this paper, it is verified on the datasets of MS COCO and Visual Genome. MS COCO is widely used in the field of image captioning. The training set, verification set, and test set contain 82,783, 40,775, and 40,504 images, respectively, with 5 descriptions for each image. To get the keyword input of the pretrained model, we use Faster RCNN to extract the entities of each image and obtain the keyword information. For the verification of the experimental effect, we follow Karpathy's segmentation method and divide the data into 113.2K/5K/5K [39]. Visual Genome includes 108,077 images, and each image contains 35 objects on average, 26 attributes, and 21 relationships among objects. The region descriptions of the dataset are marked, with each region having a bounding box. The verification and testing methods are the same as those of using MS COCO.

#### 4.1.2. Evaluation Metrics

We use the standard automatic evaluation metrics to evaluate the quality of image captions, including SPICE [40], CIDEr [41], METEOR [42], ROUGE-L [43], and BLEU [44]. B@ (1–4) stands for BLEU (1–4), and *M*, *R*, *C*, and *S* stand for METEOR, ROUGE-L, CIDEr, and SPICE, respectively.

#### 4.1.3. Implementation Details

Our backbone network uses the BERT base and is initialized using UNILM parameters. The pretrained Faster RCNN is used to extract the category information in an image as the keywords and the pretrained ResNet101 to extract the global image features as the visual reference input. Specifically, the keywords extracted by Faster RCNN are transformed into the word vector of 768, and the image information extracted by ResNet101 is obtained by using two VPNs; the image features are transformed from 2,048 dimensions to 768 dimensions. In the training process, our epoch length is set as 10, the learning rate is 0.00001, the batch size is 16, and the Adam optimizer [45] is used to progressively adjust the learning rate. In the inference phase, we use a beam search with a beam size of 3 if not specifically specified. We train our model using the standard cross-entropy loss; we minimize the following cross-entropy loss:

(17) $$\begin{array}{c} L\left(\theta \right)=-\sum_{t=1}^{T}\text{log}\left(p_{\theta }\left(y_{t}|y_{1:t}\right)\right), \end{array}$$

where  *y*_1:*t*_ is the true captioning, and *θ* is the model parameter. Within the scope of our calculation, we have carried out experiments on epoch, batch size, beam size, learning rate, etc. The settings described above are under optimal conditions.

### 4.2. Comparisons with State of the Art

#### 4.2.1. Image Captioning

To comprehensively evaluate the performance of VTR-PTM, we compare our model with two types of the existing methods on MS COCO. (1) No pretraining models are used. The methods introduce attention or graph network in the structure of encoder and decoder, such as ADAPTIVE [24], UP-DOWN [45], CAVP [46], SGAE [26], and ASG [27]; (2) pretraining models are used. The methods are based on multilayer transformers, such as ORT [47], AOANET [48], NG-SAN [29], and U-VLP [7]. The evaluation results are shown in Table 1. VTR-PTM outperformed all the baselines on multiple metrics, except that C and S are 130.2 and 28.5, which are lower than 204.2 and 42.1 of ASG [27].

**Figure 4:**
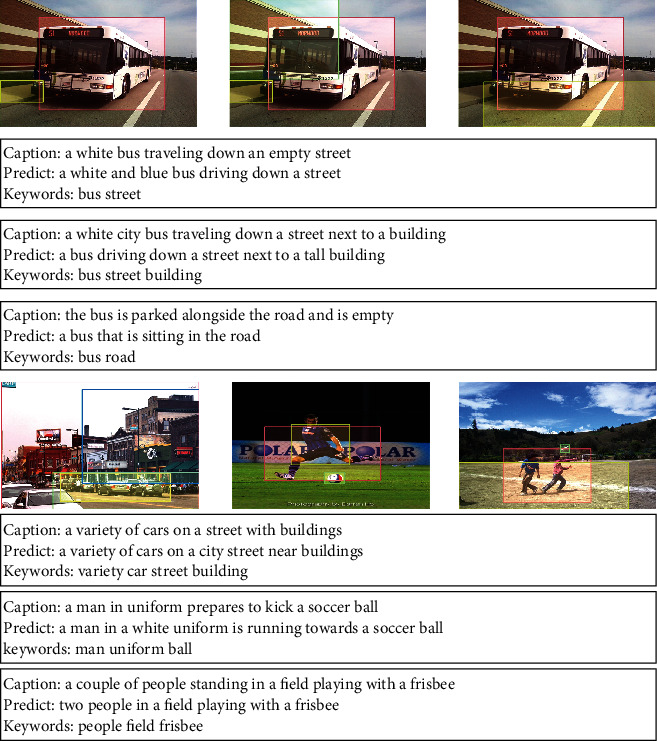
Visual presentation of image captions and corresponding visual areas on MS COCO. We use Faster RCNN to detect the objects in images and generate the corresponding keywords. In the prediction captions, we have highlighted the keywords in the color font.

We visualize the results in the test set (see Figure 4). Line 1: the same image gets different keywords by object detection. Lines 2, 3, and 4: different image captions are generated by keywords in Line1. Line 5: different images get different keywords through object detection. Line 6, 7, and 8: different image captions are generated according to the keywords and image features in Line 5. VTR-PTM generated accurate captions according to keywords and image information. In terms of syntax and content, we have achieved promising results. In the first line, three different captions are generated for the same image, and the results are consistent with the image content, indicating that VTR-PTM can generate diverse expressions.

#### 4.2.2. Dense Image Captioning

The dense image captioning is to generate the captions of different regions in an image. Relatively speaking, it means generating shorter sentences and more concrete caption content. We test the performance of our model on Visual Genome. Each image of the dataset contains the caption of 35 objects, and the results are shown in Table 2. VTR-PTM beats all the baselines substantially in all the metrics, except that C is 185.9, which is lower than 202.4 of ASG [27]. Our method is relatively more flexible because ASG needs to control the number of entities and then generate captions.

**Figure 5:**
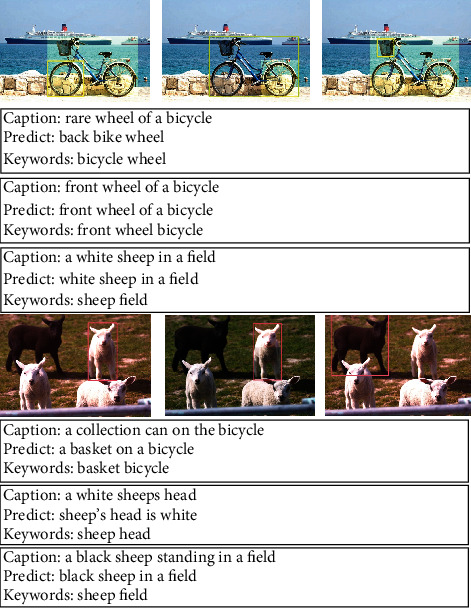
A visual display of the generated captions and the corresponding visual regions on Visual Genome.

We show some of the visualization results in Figure 5. Line 1 and Line 5: different regions in the same image get different keywords by object detection. Lines 2, 3, 4 and 6, 7, 8: image captions are generated for different regions based on keywords in Lines 1 and 3. In the first line of images, our model generates accurate captions for bicycles, wheels, etc.

### 4.3. Ablation Study

#### 4.3.1. Impact of Different References

To analyze the effects of different references, we compare three different methods that are as follows: (1) only the visual reference section is taken as input, and the keywords are set to an empty string, which is called VTR-PTM0, and the corresponding training loss is loss0; (2) only the keywords extracted are taken as input, and the visual reference is set to an empty string, which is called VTR-PTM1, and the corresponding training loss is loss1; and (3) the dual-stream input is taken, which is called VTR-PTM2, and the corresponding training loss is loss2. Experiments are carried out on MS COCO, and the results are shown in Table 3.

**Figure 6:**
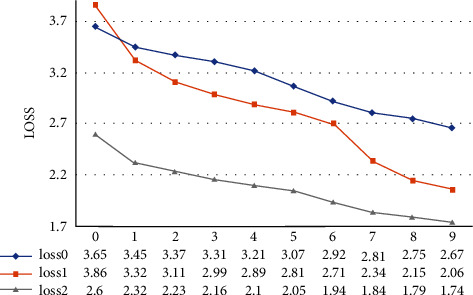
Changes of loss scores of different input methods during training. The abscissa is the value of the epoch, and the ordinate is the value of the loss.

VTR-PTM2 is significantly better than VTR-PTM0 and VTR-PTM1 alone. Although VTR-PTM1 is better than VTR-PTM0, VTR-PTM0 still plays a certain role, which also proves that the visual reference embedding proposed here is effective. We show the training losses under the same conditions for three different input methods (see Figure 6). Training losses show the same downward trend. However, the convergence trend is VTR-PTM2 > VTR-PTM1 > VTR-PTM0, and the final losses are 1.74, 2.06, and 2.67, respectively. It can be seen that the input of the two parts has a certain effect. When the pretraining model selects features based on text features, image features can help the model further learn and express according to the image content.

#### 4.3.2. Impact of Different Coding Methods

**Figure 7:**
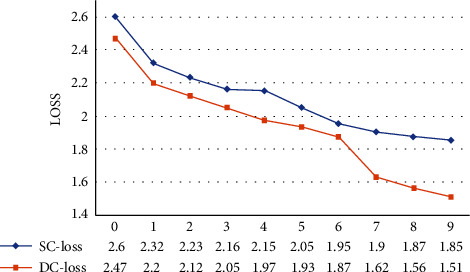
Changes in loss scores of different coding methods during training. SC-loss represents the training loss of VRN-SC. DC-loss represents the training loss of VRN-DC.

To study the influence of the reference features generated by different coding methods on the results, we design two coding methods: (1) single-channel network VRN-SC and (2) dual-channel network VRN-DC. The results are shown in Table 4. VRN-DC is superior to VRN-SC in all the evaluation metrics. In a transformer, *g* and *b* have different functions in the normalization layer. *g* is the scaling of the features, *b* is the bias of the features. Therefore, in the process of training, the influence on model learning is also different. The training loss is shown in Figure 7. During the training process, the loss decreases almost uniformly, and the final loss of VRN-SC is 1.85 and that of VRN-DC is 1.51. This indicates that reference embedding for *g* and *b* in layer normalization requires separate coding.

#### 4.3.3. Impact of Different Initialization Methods

To verify the influence of different initializations on the result of the model, we set three different methods, i.e., (1) random initialization of the model; (2) initialization with BERT parameters; and (3) initialization with UNILM parameters. The results are shown in Table 5.

The results show that using UNILM parameters to initialize our model is better than using random initialization or BERT parameter initialization. Although UNILM does not specifically train image captions, the three mask designs contain to include the design of sequence generation. This is more conducive to the learning process of our model.

## 5. Conclusion

In this paper, we propose a novel pretraining model VTR-PTM for image captioning. We design the dual-stream input mode of image reference and text reference and use two different mask modes (bidirectional and sequence to sequence) to realize the VTR-PTM suitable for generating tasks. To the best of our knowledge, VTR-PTM is the first reported pretraining model to use visual-text reference in the learning process. We have verified the effectiveness of the proposed method on the datasets of image captioning (MS COCO and Visual Genome). Compared with the state-of-the-art literature, substantial improvements are observed in multiple evaluation indices. The corresponding ablation experiments also show the effectiveness of the proposed model. In addition, our model can also be extended to audio-text or video-text tasks. In future research, we will try to combine semisupervised learning and pretraining models, which may bring better results for image captioning.

## Figures and Tables

**Table 1:** Comparisons with state-of-the-art single-model approaches on MSCOCO karpathy test split.

Approach	B@1	B@2	B@3	B@4	*M*	*R*	*C*	*S*
ADAPTIVE [24]	74.8	58.4	44.4	33.6	26.4	55.0	104.2	19.7
UP-DOWN [45]	80.2	64.1	49.1	36.3	27.7	56.9	120.1	21.4
CAVP [46]	80.1	64.7	50.0	38.6	28.3	58.9	126.3	21.6
SGAE [26]	80.6	65.0	50.1	39.0	28.4	58.9	129.1	22.2
ORT [47]	80.8	—	—	38.6	28.7	58.4	128.3	22.6
AOANET [48]	81.0	65.8	—	38.9	29.2	58.8	129.8	22.4
U-VLP [7]	—	—	—	39.5	29.3	—	129.3	23.2
NG-SAN [29]	80.8	65.4	50.8	39.9	29.3	59.2	132.1	23.3
ASG [27]	—	—	—	23.0	24.5	50.1	204.2	42.1
VTR-PTM (ours)	82.9	67.3	53.4	40.9	30.9	61.5	130.2	28.5

**Table 2:** The performance of the published state of the art and our model on the test sets of Visual Genome. In dense image captioning, the model receives a single image and generates a set of regions, each annotated with confidence and a caption.

Approach	B@4	*M*	*R*	*C*	*S*
ST [20]	11.1	17.0	34.5	139.9	31.1
UP-DOWN [45]	10.9	16.9	34.5	139.4	31.4
ASG [27]	17.6	22.1	44.7	202.4	40.6
VTR-PTM (ours)	20.5	27.8	45.3	185.9	50.9

**Table 3:** Comparison of the results generated by VTR-PTM in different input modes on MS COCO.

Approach	B@1	B@2	B@3	B@4	*M*	*R*	*C*	*S*
VTR-PTM0	71.1	55.4	40.2	29.6	24.3	51.5	100.5	20.1
VTR-PTM1	80.2	65.4	52.3	39.5	29.2	58.3	128.6	27.3
VTR-PTM2	82.9	67.3	53.4	40.9	30.9	61.5	130.2	28.5

**Table 4:** Comparison of the results generated by the visual reference network of VTR-PTM in single-channel and dual-channel coding on MSCOCO.

Approach	B@1	B@2	B@3	B@4	*M*	*R*	*C*	*S*
VRN-SC	81.9	67.1	53.2	40.7	30.3	61.0	129.7	28.2
VRN-DC	82.9	67.3	53.4	40.9	30.9	61.5	130.2	28.5

**Table 5:** Comparison of the results generated by VTR-PTM in different initialization methods on MS COCO.

Approach	B@1	B@2	B@3	B@4	*M*	*R*	*C*	*S*
VTR-PTM from scratch	80.3	63.2	50.9	37.7	28.5	56.9	123.4	25.8

VTR-PTM from BERT	81.5	66.4	52.7	38.6	29.5	58.3	125.6	27.8

VTR-PTM from UNILM	82.9	67.3	53.4	40.9	30.9	61.5	130.2	28.5

## Data Availability

The data used to support the findings of this study are available from the corresponding author upon request.
